# The roles of EGF and Wnt signaling during patterning of the *C. elegans *Bγ/δ Equivalence Group

**DOI:** 10.1186/1471-213X-9-74

**Published:** 2009-12-31

**Authors:** Adeline Seah, Paul W Sternberg

**Affiliations:** 1 HHMI and Division of Biology, California Institute of Technology, Pasadena, CA 91125, USA

## Abstract

**Background:**

During development, different signaling pathways interact to specify cell fate by regulating transcription factors necessary for fate specification and morphogenesis. In *Caenorhabditis elegans*, the EGF-Ras and Wnt signaling pathways have been shown to interact to specify cell fate in three equivalence groups: the vulval precursor cells (VPCs), the hook competence group (HCG) and P11/12. In the VPCs, HCG and P11/12 pair, EGF and Wnt signaling positively regulate different Hox genes, each of which also functions during fate specification. In the male, EGF-Ras signaling is required to specify the Bγ fate within the Bγ/δ equivalence pair, while Notch signaling is required for Bδ fate specification. In addition, TGF-β signaling by *dbl-1/dpp* controls *ceh-13/labial/Hox1* expression in Bγ.

**Results:**

We show that EGF-Ras signaling is required for Bγ expression of *ceh-13/labial/Hox1*. The transcription factors *lin-1/ETS* and *lin-31/Forkhead*, functioning downstream of the EGF pathway, as well as *sur-2/MED23* (a component of the Mediator complex) also control *ceh-13* expression in Bγ. In addition, our results indicate that *lin-44/Wnt*, *mom-2/Wnt* and *lin-17/Fz* are necessary to maintain the division of Bγ along a longitudinal axis. We also show that *dbl-1/dpp* acts either in parallel or downstream of EGF pathway to regulate *ceh-13/Hox1* expression in Bγ. Lastly, we find that a *dbl-1/dpp* null mutation did not cause any vulval or P12 defects and did not enhance vulval and P12 defects of reduction-of-function mutations of components of the EGF pathway.

**Conclusions:**

*ceh-13/labial/Hox1* expression in Bγ is regulated by the EGF pathway and downstream factors *lin-1/ETS lin-31/Forkhead* and *sur-2/MED23*. Wnt signaling is required for proper Bγ division, perhaps to orient the Bγ mitotic spindle. Our results suggest that *dbl-1/dpp* is not required for VPC and P12 specification, highlighting another difference among these EGF-dependent equivalence groups.

## Background

During development, fate specification within equivalence groups (a set of cells with similar potential) often requires extracellular cues provided by surrounding cells [1-5]. The response elicited by a particular signaling pathway is context-specific: the fate acquired by a cell depends on its developmental history (i.e., the genes expressed by a cell) as well as the presence of other external signals. One mechanism by which signaling pathways specify fate is by regulating master control genes that initiate expression of a battery of genes required for a particular fate. Hox genes are a class of master regulators that pattern the anterior-posterior axis of metazoans during embryogenesis. In *C. elegans*, there is accumulating evidence that different Hox genes are upregulated by Wnt and EGF-Ras signaling in different equivalence groups.

EGF and Wnt signaling act together to specify fates within three different equivalence groups in *C. elegans*: the vulval precursor cells (VPCs), the hook competence group (HCG) and the P11/12 group [6-10]. Each of these equivalence groups involves the patterning of Pn cells. During the first larval (L1) stage, each postembryonic Pn (n = 1, 2, 3,..., 12) precursor cell is positioned along the anterior-posterior axis on the ventral epithelium and divides to produce an anterior (Pn.a) and a posterior daughter (Pn.p). The P11/12 equivalence group is found in both hermaphrodites and males, and EGF and Wnt signaling are required to specify the P12 fate, which is the 1° fate. In hermaphrodites, the central Pn.p cells, P3-8.p, comprise the VPCs, which can each adopt a 1°, 2° or 3° vulval fate. The EGF-Ras pathway induces the 1° VPC fate while Wnt signaling plays a minor role in induction. In males, the posterior Pn.p cells, P9-11.p, form the HCG that gives rise to the hook (a male reproductive structure involved in vulva location behavior). Similar to the VPCs, there are three HCG fates: 1°, 2° or 3°. However, in contrast to vulval development, Wnt signaling is the major inductive signal during hook development, specifying the 1° and 2° HCG fates [11]. A role for EGF-Ras signaling in HCG specification is only observed when Wnt signaling is compromised. In addition, LIN-12/Notch signaling specifies both the 2° VPC and 2° HCG fates by lateral signaling [12,13].

Different Hox genes are required to specify vulval and P12 fates downstream of the EGF and Wnt pathways. Specifically, *lin-39/SexcombsReduced/Hox5 *is upregulated in the VPCs by EGF and Wnt signaling, while *egl-5/Antennapedia/Ultrabithorax/Hox6/8 *is expressed in P12 and upregulated by EGF, and most likely Wnt signaling, in P12.pa (a descendant of P12) [8,9,14]. Overexpression of *lin-39 *or *egl-5 *is also partially sufficient to specify vulval or P12 fates, respectively. Although a role for MAB-5/Antennapedia/Ultrabithorax/Hox6/8 has not been shown in the HCG, *mab-5 *is expressed in the HCG [15] and is regulated by Wnt signaling (Seah, A., and Sternberg, P.W., unpublished observation). In addition, increased Notch signaling in *lin-12(gf) *males results in P(3-8).p acquiring vulval fates and P(9-11).p adopting hook fates, implying that P(3-8).p and P(9-11).p have different propensities to generate vulval and hook lineages, respectively [12]. Overexpression of MAB-5 in *lin-39(rf) *hermaphrodites also causes P(5-7).p to display hook-like features [16]. Taken together, these observations suggest, that similar to vulval and P12 development, a Hox gene (*mab-5*) may be required to specify HCG fates. A fourth Hox gene, *ceh-13/labial/Hox1*, is expressed in another equivalence group that requires EGF signaling for fate specification: the γ/δ pair generated by the B cell, a male-specific blast cell.

**Figure 1:**
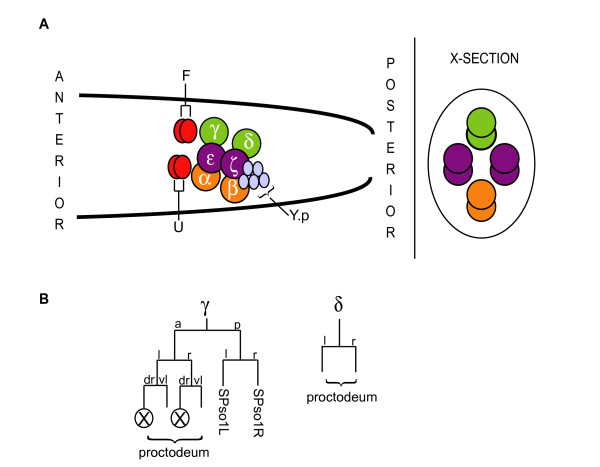
**The Bγ/δ equivalence group during development**. (A) Arrangement of the B.a progeny during the mid-L3 stage, adapted from Chamberlin and Sternberg (1993). Left lateral view and cross section. (B) Cell division patterns of Bγ and Bδ, adapted from Sulston et al. (1980). Circled crosses indicate pairs of cell in which the left or right cell dies.

The B cell gives rise to the male copulatory spicules [6,17]. B.a generates eight cells grouped into four anterior-posterior pairs that form the γ/δ, α/β and the two ε/ζ equivalence groups (Fig. 1A). Each cell type has a distinct division pattern. In particular, Bγ divides in a longitudinal fashion (at about a 45° angle to the anterior-posterior, A/P, axis where Bγ.a is dorsal to Bγ.p) and produces six progeny where one dies, while Bδ divides in a transverse fashion once to produce two progeny. Of the five remaining γ progeny, two are neuronal support cells and three are proctodeal cells; both Bδ progeny are proctodeal cells. Several findings indicate that EGF signaling specifies the anterior cell fate of each equivalence pair. Ablation of the male-specific blast cells, U and F, which are one source of anterior *lin-3/EGF*, can cause the anterior cell to adopt the posterior fate [18-20]. In addition, reduction-of-function (rf) mutations in *lin-3/EGF*, *let-23/EGFR*, *sem-5/Grb2*, *let-60/Ras *and *lin-45/Raf *cause anterior-to-posterior fate transformations within each equivalence group [20]. Conversely, excessive EGF signaling due to ectopic expression of the EGF domain using a heat-shock transgene or a *lin-15(null) *mutation causes the posterior cell to acquire the anterior fate. Fate transformations in these experiments were assayed based on the number of progeny generated by each fate and the orientation of the first division of the Bγ/δ pair after induction (Fig. 1B).

The Bγ/δ pair was characterized in further detail by the ablation of the posterior daughter of Y, another male-specific blast cell, which indicated a role for Y.p in promoting the posterior fate, Bδ [19]. In addition, when U and F are absent or when U, F and Y.p are absent, increased LIN-12/Notch signaling in *lin-12(gf) *males causes Bγ-to-δ fate transformations [20]. These results suggest that LIN-12/Notch is sufficient to specify the Bδ fate in the absence of Y.p. Conversely, reduced LIN-12/Notch signaling in *lin-12(null) *males resulted in Bδ-to-γ fate transformations. However, since Y.p is absent in *lin-12(null) *males, it is not possible to establish whether Y.p is sufficient to specify the Bδ fate in these mutants. In the absence of U, F and Y.p, the Bγ/δ equivalence pair is still able to express the Bγ and Bδ fates, suggesting that other external cues act to specify these fates. Furthermore, reduced EGF signaling did not cause a Bγ-to-δ fate transformation in all animals: partial fate transformations were observed in which the presumptive Bγ cell either divided in a wild-type, longitudinal fashion but produced four progeny (less than the wild-type number of six progeny) or divided in a transverse fashion (Bδ-like) but produced more than two progeny (Bγ-like). Unfortunately, it is not possible to determine Bγ fate specification in mutants carrying null alleles of EGF signaling pathway components because EGF signaling is required for viability at an earlier larval stage.

Stoyanov et al. (2003) reported that *ceh-13/labial *was expressed in Bγ and that expression required *dbl-1/dpp/TGF-β*, *sma-2/R-Smad*, *sma-3/R-Smad *and *sma-4/Co-Smad *-- components of the TGF-β pathway that also regulates the Sma/Mab pathway in *C. elegans *[21-24]. Moreover, in *Drosophila*, the TGF-β, EGF and Wnt pathways regulate *labial *expression during midgut morphogenesis [25-27]. Therefore, we wished to investigate whether EGF and Wnt signaling also regulate *ceh-13/labial *expression. And conversely, since the TGF-β pathway was reported to regulate *ceh-13/labial *expression, we also examined whether TGF-β signaling is involved in VPC, HCG and P12 specification.

Here, we show that the EGF pathway is required for the expression of *ceh-13/labial/Hox1 *in Bγ. In addition, we find that *lin-1/ETS *and *lin-31/Forkhead *(transcription factors which act downstream of EGF signaling during vulval development) and one of the components of the Mediator complex, *sur-2/MED23*, are required for *ceh-13/Hox1 *expression in Bγ. We also provide evidence that *lin-44/Wnt*, *mom-2/Wnt *and *lin-17/Fz *control the Bγ division axis but are not required for *ceh-13 *expression. Our results indicate that EGF and TGF-β signaling by the *C. elegans dpp/BMP *ortholog, *dbl-1*, specify the Bγ fate and that TGF-β signaling likely acts downstream or in parallel to the EGF pathway. By contrast, we show that *dbl-1/TGF-β *signaling appears to have no role in VPC and P12 specification. Since the other equivalence groups also use the EGF and Wnt pathways, TGF-β signaling may contribute to the specificity of the Bγ fate.

## Results

### EGF-Ras signaling positively regulates transcription of *ceh-13/labial/Hox1 *in Bγ

**Figure 2:**
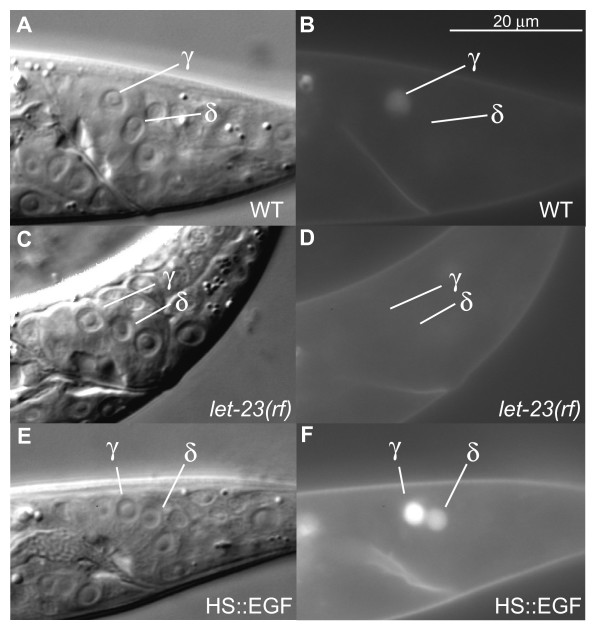
**EGF signaling is necessary and sufficient for *ceh-13*::GFP expression in the Bγ/δ pair (A-B) Mid-L3**. Wild-type *ceh-13*::GFP expression was only observed in Bγ. (C-D) Mid-L3 *let-23(rf) *males. *ceh-13*::GFP was not expressed in Bγ. Similar observations were made in *sem-5(rf) *and *let-60(rf) *mutants. (E-F) Mid-L3. Increased EGF signaling in heat-shocked HS::EGF males caused ectopic *ceh-13*::GFP expression in Bδ, in addition to wild-type Bγ expression. Similar observations were made in *lin-15(lf) *and *let-60(gf) *mutants. Left lateral views. Scale bar in B, 20 μm for A-F.

To study *ceh-13/Hox *regulation by EGF/Ras signaling, we utilized an integrated transcriptional GFP reporter, *syIs145*, that contains about 8 kb upstream sequence and the first and second exon of *ceh-13 *fused to GFP. In s*yIs145 *males, *ceh-13*::GFP was observed in Bγ in 100% of animals by the mid-L3 stage (Fig. 2A-B, Table 1). First, we ablated the U and F male-specific blast cells that are required for proper Bγ fate specification and express the *lin-3/EGF *ligand [18,19]. In the majority of males in which the U and F cells were killed, we found that *ceh-13*::GFP was absent in Bγ (Table 1). Because null alleles of EGF signaling mutants cause larval lethality [28-30], we used *let-23/EGFR*, *let-60/Ras *and *sem-5/Grb-2 *reduction-of-function (rf) mutations to determine if EGF signaling is required for *ceh-13 *expression. We observed a significant decrease of *ceh-13*::GFP expression in Bγ in all strains (Fig. 2C-D, Table 1). Therefore, EGF/Ras signaling positively regulates *ceh-13 *transcription in Bγ.

**Table 1:** Reduced EGF signaling causes loss of *ceh-13*::GFP expression

**Genotype**^a^	n	*ceh-13*::GFP ^b ^in Bγ (%)
Intact, wild type	41	100
Mock ablated, wild type	3	100
U^-^F^-c^, wild type	8	12.5***
*let-60(rf)/Ras*	42	57.1***
*let-23(rf)/EGFR*^d^	20	55***
*sem-5(rf)/Grb-2*	30	26.7***

***p < 0.0001; Fisher's Exact Test. The p values for all genotypes were calculated as compared to the Intact, wild-type genotype, except for U and F ablated wild-type males which were compared to mock ablated wild-type males.

^a ^The alleles used were *let-23(sy97)*, *let-60(n2021) *and *sem-5(n1619)*. All strains contained *him-5(e1490)*.

^b ^All strains examined carried the integrated *ceh-13*::GFP transgene, *syIs145*.

^c ^F and U were ablated in these animals.

^d ^*ceh-13*::GFP expression was much dimmer as compared to wild-type expression in 7 of the 11 *let-23(rf) *males that had expression in γ.

Since activation of EGF/Ras signaling has been shown previously to be sufficient to induce a Bδ-to-γ fate transformation, we hypothesized that increased EGF signaling would cause ectopic expression of *ceh-13*::GFP in Bδ. We tested this hypothesis using several different methods. One method was to use a transgenic construct that places the *lin-3/EGF *cDNA under control of a heat-shock promoter to generate ectopic expression of *lin-3/EGF *[31]. We found that 60% of heat-shock treated animals carrying the HS::LIN-3C construct had abnormal *ceh-13*::GFP expression in Bδ (Fig. 2E-F, Table 2). We also made use of a *let-60 *gain-of-function (gf) allele, *n1046*, which constitutively activates Ras signaling. We found that in 18% of *let-60(n1046) *animals, *ceh-13*::GFP was ectopically expressed in Bδ (Table 2). In addition, a loss-of-function (lf) mutation in the *lin-15 *locus, which normally acts to antagonize the EGF/Ras pathway [32,33], caused *ceh-13*::GFP expression in Bδ (Table 2). Our results suggest that increased EGF signaling is capable of promoting *ceh-13*::GFP expression in Bδ and that *ceh-13*::GFP expression is an early indicator of the Bγ cell fate.

**Table 2:** Increased EGF signaling causes ectopic *ceh-13*::GFP expression

Genotype^a^	n	*ceh-13*::GFP ^b ^in Bδ (%)
Wild type	41	0
Wild type, 1 hr heat-shock	25	0
*lin-15(lf)*	38	18.4**
Integrated HS::EGF, 1 hr heat-shock	30	86.7***
*let-60(gf)*/Ras	28	17.9*

***p < 0.0001, **p < 0.005, *p < 0.05; Fisher's Exact Test. The p values for all genotypes were calculated as compared to wild-type males, except for heat-shocked HS::EGF worms that were compared to heat-shocked wild-type males.

^a ^The alleles used were *lin-15(e1763) *and *let-60(n1046)*. The integrated HS::EGF transgene *syIs197 *was used. All strains contained *him-5(e1490)*.

^b ^The integrated *ceh-13*::GFP transgene *syIs145 *was used.

Therefore, in addition to the number of progeny generated and the orientation of the first division, the Bγ fate is characterized by lineage-specific gene expression (i.e. *ceh-13 *expression).

### *lin-1/ETS, lin-31/Forkhead *and *sur-2/Mediator *function during Bγ specification

Since we had found that *ceh-13 *transcription is controlled by EGF signaling, we investigated whether *lin-1/ETS *and *lin-31/Forkhead*, transcription factors known to mediate other EGF-Ras signaling events such as vulval development [34-36], also regulate *ceh-13 *expression. A role for either transcription factor during Bγ specification has not previously been identified. In addition, we also tested if *sur-2/MED23 *(a component of the Mediator complex), which has been shown to act downstream of Ras, regulated *ceh-13/hox1 *expression [37].

#### *lin-1/ETS* has both a positive and negative role in Bγ specification

Members of the ETS domain transcription factor family are downstream effectors of Ras signaling in many organisms [38]. *lin-1 *is the *C. elegans *ETS homolog and has both a positive and a negative role downstream of EGF-Ras signaling in vulval development, excretory duct cell specification, P12 specification and hook development [35,39,40]. Several results suggest that *lin-1 *functions in a similar manner during Bγ specification. First, we examined the effects of severe reduction-of-function and gain-of-function mutations on *ceh-13*::GFP expression. The *n1790gf *and *n1761gf *alleles cause strong abnormal vulva and larval lethality phenotypes by severely reducing the negative regulation of LIN-1 by the EGF pathway [41]. We observed a loss of *ceh-13*::GFP expression in Bγ in *lin-1(rf)*, indicating that there is a positive requirement for LIN-1 for *ceh-13/Hox1 *expression in Bγ (Table 3). Furthermore, a loss of *ceh-13*::GFP expression in Bγ in *lin-1(gf) *mutants was observed, suggesting that LIN-1 has a negative effect on *ceh-13/Hox1 *expression in Bγ (Table 3). In addition, we found that *ceh-13*::GFP was ectopically expressed in Bδ in *lin-1(rf) *males, which suggests that LIN-1 inhibits Bδ from expressing the Bγ fate (Table 3). Therefore, LIN-1 positively and negatively regulates transcription of *ceh-13*.

**Table 3:** *lin-1*, *lin-31 *and *sur-2 *regulate *ceh-13*::GFP expression

Genotype^a^	n	*ceh-13*::GFP^b ^in Bγ (%)	*ceh-13*::GFP in Bδ (%)
Wild type	41	100	0
*lin-1(e1777rf)*	34	85.3*	41.2***
*lin-1(n1761gf)*	30	76.7**	0
*lin-1(n1790gf)*	30	30***	0
*lin-31(bx31lf)*	33	63.6***	0
*lin-31(n301lf)*	32	87.5*	0
Int HS::*lin-3*^b^	30	100	86.7***
*lin-1(n1790gf)*; Int HS::*lin-3*^b^	15	33.3***	0***
*lin-31(n301lf)*; Int HS::*lin-3*^b^	30	83.3	26.7***
*sur-2(sy260rf)*	17	64.7**	0

***p < 0.0001, **p < 0.005, *p < 0.05; Fisher's Exact Test. All p values were calculated as compared to wild type males, except for p values for the *lin-1(n1790gf)*; Int HS::*lin-3 *and *lin-31(n301lf)*; Int HS::*lin-3 *strains which were each compared to the Int HS::*lin-3 *strain.

^a ^All strains contained *him-5(e1490) *and the integrated *ceh-13*::GFP transgene *syIs14*5.

^b ^The integrated HS::EGF transgene *syIs197 *was used.

Based on the other criteria for fate specification (i.e., the number of progeny and the axis of the first division), the requirement of *lin-1 *during Bγ fate specification appears to be minor and may be redundant with other factors because the Bγ lineage is normal in all *lin-1(e1777rf) *animals observed (n = 7; H. Chamberlin, personal communication). In addition, the *lin-1(gf) *mutation is not sufficient to cause a complete Bγ-to-δ transformation: we observed that Bγ divided in a wild-type longitudinal manner in *lin-1(n1790gf) *males (n = 10), and Bγ divided more than once in four of these *lin-1(gf) *males. However, Bδ in the majority of the seven *lin-1(e1777rf) *animals in which lineages were followed acquires a Bγ-like fate (6/7), indicating that *lin-1 *inhibits Bδ from expressing the Bγ fate.

To confirm that *lin-1 *lies downstream of the EGF signal in Bγ and Bδ, we tested whether a *lin-1(gf) *mutation could suppress the effects of increased EGF signaling. We found that *ceh-13*::GFP expression in heat-shocked *lin-1(n1790gf)*; HS::EGF animals was similar to *lin-1(n1790gf) *single mutants (Table 3), indicating epistasis of *lin-1 *over excessive LIN-3/EGF in Bγ and Bδ. Therefore, our results suggest that *lin-1 *acts downstream of or in parallel to the EGF pathway.

#### *lin-31/Forkhead* has a positive role in Bγ specification

*lin-31 *belongs to the Forkhead family of transcription factors and like *lin-1/ETS *also acts positively and negatively downstream of the EGF-Ras pathway in vulval development [36]. However, unlike *lin-1/ETS*, *lin-31/Forkhead *was reported to be specific to EGF/Ras signaling during vulval development and was not thought to act during the specification of the B equivalence groups even though LIN-31 is expressed in Bγ, Bδ and Bε [34]. We determined that *lin-31 *is required neither for 1° and 2° hook fate specification nor for P12 development (n = 7 and n = 32, respectively). However, we found that LIN-31 is required to positively regulate *ceh-13 *transcription: *ceh-13*::GFP expression in Bγ was absent in about 36% of *lin-31(bx31*) and 12% of *lin-31(n301) *(Table 3). *n301 *is a null allele of *lin-31 *[42], while *bx31 *is presumably a null allele of *lin-31 *[43]. Since we never observed abnormal *ceh-13*::GFP expression in Bδ in *lin-31 *mutants, it appears that *lin-31 *only has a positive role during Bγ specification. Similar to *lin-1/ETS*, *lin-31 *also lies downstream of the EGF signal because *lin-31(n301) *is able to suppress the effects of increased EGF signaling due to ectopic expression of the EGF ligand (Table 3). Therefore, *lin-31 *is not a vulval-specific effector of EGF/Ras signaling.

In addition to controlling *ceh-13 *expression, we found that LIN-31 also affects the axis of the first division of Bγ. In about 90% of *lin-31(bx31lf) *(n = 30) and *lin-31(n301lf) *(n = 33) mutants, which had wild-type *ceh-13 *expression, we observed that Bγ divided along a transverse axis, similar to Bδ, rather than along a longitudinal axis (For both strains: p < 0.0001; Fisher's Exact test). The division plane of Bγ in *lin-31(lf) *mutants strongly resembles that of Bδ, distinct from the abnormal Bγ division phenotype observed in Wnt mutants (discussed in the following section), suggesting that effects on the axis of division in *lin-31(lf) *mutants are probably caused by fate specification defects. However, we found that Bγ divided more than once in five *lin-31(n301lf) *animals, indicating that a complete Bγ-to-δ transformation did not occur; in the wild-type male, Bδ divides only once. Our results suggest that downstream of the EGF pathway, other factors, such as LIN-1/ETS, act with LIN-31/Forkhead to specify the Bγ lineage.

#### *sur-2/Mediator complex subunit 23(MED23)* upregulates *ceh-13/Hox1* expression

A number of other transcription factors have been shown to act downstream or in parallel to the EGF-Ras pathway in *C. elegans *during one or more of the following events: vulval development, P12 specification and larval viability. Mutations in these factors cause phenotypes similar to those caused by mutations in components of the EGF signaling pathway. Of the factors we tested (Additional File 1), only *sur-2/MED23*, which has been shown to act downstream of Ras [37], regulated *ceh-13/hox1 *expression

Using both *ceh-13 *expression and the division axis of Bγ to assay fate, we found that the *sur-2(sy260rf) *mutation caused defects in Bγ fate specification: *ceh-13*::GFP was not expressed in Bγ in 35% of *sur-2(sy260rf) *males (Table 3, p = 0.0003; Fisher's Exact Test). In addition, Bγ divided in a transverse Bδ -like manner in 21% of *sur-2(sy260rf) *males (n = 14, p = 0.0275; Fisher's Exact Test).

### Wnt signaling controls Bγ division axis

As Wnt signaling has been shown to act together with EGF signaling to specify vulval fates and P12 fate by regulating the Hox genes [8,9], we decided to test whether the Wnt signaling pathway also specified the Bγ fate. There are five Wnt-like genes in the *C. elegans *genome -- *lin-44*, *egl-20*, *mom-2*, *cwn-1 *and *cwn-2 *-- and we first examined *ceh-13*::GFP expression to assay Bγ fate specification in Wnt mutants. None of the Wnt single or double mutants examined in Table 4 displayed defects in *ceh-13*::GFP expression (data not shown). However, we observed defects in the first division of Bγ in 22.7% of *mom-2(lf) *homozygotes derived from *mom-2(lf)/+ *hermaphrodites and in 44% of *lin-44(lf) *males (Table 4). Of the animals that exhibited a defect, Bγ divided obliquely (at a wild-type 45° angle to the A/P axis but in a slightly transverse manner) in seven *lin-44(lf) *males and all *mom-2(lf) *males. Bγ in three *lin-44(lf) *males divided transversely, similar to δ; the angle of division of Bγ in the A/P axis in the remaining 5 *lin-44(lf) *males was either more or less than the wild-type 45° and also slightly transverse. For example, the posterior daughter was slightly dorsal in relation to the anterior daughter instead of the opposite (as in the wild type). Our data suggest abnormal mitotic spindle orientation of Bγ occurred in these animals.

**Table 4:** Wnt signaling controls spindle orientation in Bγ

Genotype^a^	n	Bγ division plane
		
		Abnormal (L/R) (%)
Wild type	30	0
**Wnts**		
*cwn-2(ok895lf)*	30	0
*egl-20(hu120rf)*	27	0
*cwn-1(ok546lf); egl-20(n585rf)*	33	0
*lin-44(n2111lf)*	34	44.1***
*mom-2(or42lf)*	22	22.7*
**Wnt receptor**		
*lin-17(n698rf)*	33	27.3**

***p < 0.0001, **p < 0.005, *p = 0.01; Fisher's Exact Test. All p values were calculated as compared to wild type.

^a ^All strains contained *him-5(e1490) *and the integrated *ceh-13*::GFP transgene *syIs14*5.

**Figure 3:**
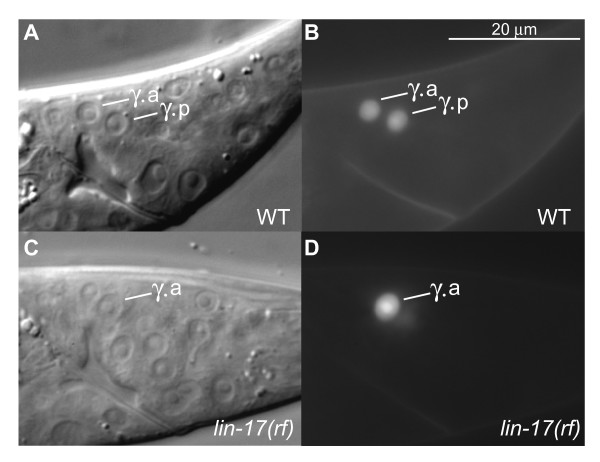
**Wnt signaling is required to maintain the division axis of Bγ**. (A-B) Mid-L3. Bγ divides along the longitudinal axis in wild-type males. (C-D) Mid-L3 *lin-17(n698rf) *male. Bγ divides in a transverse manner. Only Bγ.a can be seen in this plane and the posterior daughter of Bγ is out of focus in this picture. Left lateral views. Scale bar in B, 20 μm for A-D.

Because *lin-17/Fz *has been shown to act downstream of *lin-44/Wnt *earlier in the B lineage as well as during other developmental events, we tested if *lin-17(n698rf) *males had similar Bγ defects. Although *ceh-13::*GFP expression was wild-type in all mutants examined, we found defects in Bγ division in about a quarter of them (Table 4). Bγ divided transversely in four *lin-17(rf) *males (Fig. 3) and in 5 animals the angle of division of Bγ in the A/P axis was either more or less than the wild-type 45°. The *mom-2(or42) *defect was not as severe as the defects observed in *lin-44(lf) *or *lin-17(rf) *animals. Therefore, Wnt signaling involving *lin-44*, *mom-2 *and *lin-17 *is necessary for the Bγ division axis.

Next, we used another criteria of Bγ fate specification, the number of progeny generated, to determine if Wnt signaling is required to specify fate. Since *lin-44(lf) *mutants had the most penetrant Bγ defect, we performed lineage analysis of Bγ in *lin-44(lf) *males in which Bγ divided in a transverse Bδ-like orientation. We observed that Bγ divided more than once in all six *lin-44(lf) *males in which Bγ divided transversely, indicating that the *lin-44 *mutation does not cause a complete Bγ-to-δ fate transformation. Thus, based on *ceh-13 *expression and lineage analysis, *lin-44 *appears to only be required for Bγ to divide along the correct axis and not for Bγ fate specification. Since the *mom-2(lf) *and *lin-17(rf) *animals we examined have less severe or less penetrant defects than *lin-44(lf) *animals, it is unlikely that they will have a more severe lineage defect (i.e., fewer progeny) than *lin-44(lf) *animals.

### TGF-β pathway acts either downstream or in parallel to EGF signaling during Bγ fate specification

We have shown that similar to vulva, hook and P12 specification, EGF and Wnt signaling both affect Bγ development but they appear to have distinct roles in this latter case. Previously, TGF-β signaling was reported to play a role in Bγ specification: Stoyanov et al. (2003) reported that mutations in the TGF-β signaling components *dbl-1/dpp/TGF-β*, *sma-2/R-Smad*, *sma-3/R-Smad *and *sma-4/Co-Smad*, caused loss of *ceh-13*::GFP expression in Bγ. We wished to further investigate the role of TGF-β signaling in Bγ specification. *wk70 *is a null allele of *dbl-1 *that truncates the mature domain [21]. First, we confirmed the findings of Stoyanov et al. (2003) that *ceh-13 *expression in Bγ was abolished in *dbl-1(wk70) *males (n = 14). We also observed that in only 5 of 7 animals, Bγ divided abnormally, along a transverse axis, indicating that Bγ fate specification was not completely defective in *dbl-1(wk70) *males. This result suggests that other signaling pathways, such as the EGF pathway, likely act with DBL-1 to specify Bγ fate.

Next, to determine whether the EGF pathway acted downstream of the TGF-β pathway, we investigated whether EGF signaling was sufficient to specify the Bγ fate when TGF-β activity was reduced. Therefore, we tested whether increased EGF signaling was sufficient to induce *ceh-13*::GFP expression in a *dbl-1(null) *background because increased EGF signaling was sufficient to induce a Bδ-to-γ fate transformation [20]. We found that there was a loss of *ceh-13*::GFP expression in Bγ in all 15 heat-shocked HS::EGF; *dbl-1(null) *males examined. Our results indicate that signaling by the TGF-β ligand *dbl-1 *acts either downstream or in parallel to the EGF pathway to specify the Bγ fate.

### TGF-β signaling does not appear to be required for VPC and P12 fate specification

Since EGF signaling plays a major role during Bγ fate specification, we decided to investigate if TGF-β signaling was also required in other specification events in which the EGF pathway was the major inductive signal. If TGF-β signaling acts only during γ specification, it may contribute to the specificity of γ cell fate versus the other cell fates that require EGF signaling. Although *dbl-1(wk70) *animals exhibit wild-type vulval and P12 development (Table 5), it is possible that *dbl-1 *may only play a minor role in these specification events that could only be revealed in a sensitized background. Therefore, we next tested whether reduced TGF-β signaling would enhance the vulval and P12 defects caused by reduced EGF activity to determine whether *dbl-1/TGF-β *was required during VPC and P12 specification. Because *let-23(null) *mutations cause larval lethality, we constructed double mutants of *dbl-1(wk70) *with *let-23(rf) *or *sem-5(rf) *alleles. *sy1 *is a weak reduction-of-function allele of *let-23 *that causes vulval induction defects but no P12 defect [44]. *sy97 *is a severe reduction-of-function allele of *let-23 *that causes a completely penetrant Vul phenotype and a partially penetrant P12-to-11 transformation [9,44]. *n1779 *is a weak reduction-of-function allele of *sem-5 *that was reported previously to cause a slight Vul phenotype [30]. We found that vulval defects in *let-23(sy1)*; *dbl-1(wk70) *and *sem-5(n1779)*; *dbl-1(wk70) *double mutants were similar to *let-23(sy1) *and *sem-5(n1779) *single mutants, respectively (Table 5). These results suggest that *dbl-1 *is not required for vulval induction.

**Table 5:** *dbl-1/TGF-β *does not appear to be required for VPC or P12 specification

**Strains**^a^	Vulval Induction Index (n)	% P12→11 transformation (n)
*dbl-1(lf)*	3.0 (54)	0 (36)
*let-23(rf)*	0.27 (39)	0 (21)
*let-23(rf)*; *dbl-1(lf)*	0.31 (27)	0 (37)
*sem-5(rf)*	3.0 (81)	0 (23)
*sem-5(rf)*; *dbl-1(lf)*	3.0 (50)	0 (11)

^a ^The alleles used were *dbl-1(wk70)*, *let-23(sy1) *and *sem-5(n1779)*. All strains contained *him-5(e1490)*.

We were unable to determine if *dbl-1(wk70) *could enhance the P12 defects observed in *let-23(sy97) *animals because *let-23(sy97)*; *dbl-1(wk70) *animals were embryonic lethal. Therefore, we examined P12 fate in *let-23(sy1)*; *dbl-1(wk70) *and *sem-5(n1779)*; *dbl-1(wk70) *double mutants because although *let-23(sy1) *and *sem-5(n1779) *animals have no P12 defects, they may still provide a sensitized background in which EGF signaling is reduced in P12. Our results suggest that *dbl-1 *does not act during P12 development, as we observed wild-type P12 fates in 100% of double mutants (Table 5). However, *sy1 *and *n1779 *are hypormophic mutations, and it is possible that they do not sufficiently affect the functioning of their gene product during P12 specification.

## Discussion

We have demonstrated that the EGF and Wnt pathways act together during male Bγ development but each pathway performs different roles. EGF signaling positively regulates Hox gene *ceh-13/labial *in Bγ. This regulatory relationship is similar to vulval development and P12 specification, in which EGF signaling positively regulates the Hox genes *lin-39/Scr *and *egl-5*/*Ant/Ubx*, respectively. We also provide evidence that Wnt signaling controls the division axis of Bγ: Single or double Wnt mutants did not have defects in *ceh-13/labial *expression, but *lin-44/Wnt*, *mom-2/Wnt *and *lin-17/Fz *mutants had defects in maintaining the correct division axis of Bγ. Finally, we showed that TGF-β signaling by the *C. elegans dpp *ortholog *dbl-1 *likely acts in Bγ fate specification but neither VPC induction nor P12 specification (i.e., other EGF and Wnt regulated developmental events).

### EGF and Wnt signaling roles during Bγ development

#### EGF Pathway in Bγ Development

EGF-Ras signaling has previously been shown to specify the Bγ fate, and we showed that *ceh-13/labial *transcription is partially regulated by EGF-Ras signaling in Bγ. In addition, we found that the transcription factors *lin-1/ETS*, *lin-31/Forkhead *and *sur-2/Mediator *play roles during Bγ specification. During development, EGF signaling induces the Bγ fate by inhibiting LIN-1, which in turn inhibits the Bγ fate in the Bγ/δ equivalence group. *lin-1 *also acts to inhibit Bδ from expressing the Bγ fate because insufficient EGF signal is received by the presumptive Bδ to relieve inhibition of Bγ fate specification by *lin-1*. Our data supports other evidence that *lin-1 *[40] and *sur-2/MED23 *[37] act positively downstream of EGF signaling. Similar to our observations on *ceh-13*::GFP expression, the *lin-1(e1777rf) *allele and both *lin-1(gf) *alleles we examined have been shown to be required for *egl-17*::GFP expression in P6.p in hermaphrodites. One explanation for the apparent contradiction of *lf *and *gf *alleles having the same effect on gene expression may be that in the absence of a signal such as EGF, LIN-1 acts as an inhibitor at the promoter of the target gene, but in the presence of the signal, LIN-1 converts from an inhibitor to an activator of gene expression. The ETS protein Elk-1 has been shown to both repress and activate the same gene [45].

Previous work suggested that *lin-31/Forkhead *only functioned during vulval development downstream of EGF-Ras signaling [34]. However, our results indicated otherwise, and thus *lin-31/Forkhead *does not appear to confer specificity to EGF-Ras regulated fate specification events in *C. elegans*.

TGF-β signaling has been previously reported to be absolutely required for *ceh-13 *expression, indicating a role for TGF-β during Bγ fate specification. We confirmed those results but also observed that in some *dbl-1(null) *males, Bγ displays a wild-type axis of division. We also demonstrated that signaling by DBL-1 probably acts downstream or in parallel to the EGF pathway to specify Bγ fate.

#### WNT Pathway in Bγ Development

All Wnt single or double mutants examined had wild-type *ceh-13/labial *expression in Bγ. Because there are five Wnt genes in *C. elegans*, we were unable to definitively rule out a role for Wnt signaling in regulating *ceh-13/labial *expression. However, Bγ divided in a Bδ-like manner (transverse) in *lin-44/Wnt*, *mom-2/Wnt *and *lin-17/Fz *mutants. Furthermore, *lin-44 *and *lin-17 *Bγ defects were more severe than in *mom-2 *mutants: the axis of division was sometimes almost perpendicular to the wild-type axis. In six *lin-44(lf) *males in which Bγ divided along a transverse axis, Bγ divided more than once (characteristic of the γ lineage), indicating that Bγ did not undergo a complete Bγ-to-δ transformation in *lin-44/Wnt *mutants. One possibility is that Wnt signaling by *lin-44/Wnt*, *mom-2/Wnt *and *lin-17/Fz *acts to orient the Bγ mitotic spindle. We do not have evidence that *lin-44/Wnt*, *mom-2/Wnt *and *lin-17/Fz *are required to specify other aspects of Bγ fate.

Because LIN-44 and LIN-17 function during the orientation of the B cell division [46-48], we bypassed their requirement earlier in the lineage by using a *lin-17 *reduction-of-function allele. It was extremely difficult to find *lin-17(n671lf) *males that had wild-type B cell specification which would allow us to determine Bγ defects. By comparison, although *lin-44(n2111) *has been described as a null allele [46], we were able to find enough males in which B divided and produced a Bγ/δ pair. A different null allele of *lin-44*, *n1792*, had very few males with wild-type B specification, suggesting that there was still some gene function in *n2111 *mutants. Similarly, *mom-2(lf) *homozygotes may still have some MOM-2 activity because MOM-2 is required maternally during embryogenesis and *mom-2lf) *homozygotes examined were derived from *mom-2/+ *hermaphrodites. Therefore, we cannot exclude the possibility that sufficient gene function in each of the Wnt signaling mutants may have masked a requirement during fate specification based on our assays (i.e., number of progeny generated, *ceh-13 *expression). It is also possible that the Wnt pathway plays a role in Bγ fate which will be revealed upon reducing the activity of the right combination of Wnts, since multiple Wnts have been shown to act redundantly during other *C. elegans *developmental events e.g., [49,50].

The downstream components of Wnt/Fz signaling that control spindle orientation in the *C. elegans *embryo have been identified [51,52]. Downstream of Wnt/Fz signaling, several Dishevelled family members as well as *gsk-3/GSK-3β *align the EMS and ABar spindles. In addition, Wnt transcriptional activity mediated by *wrm-1/β-catenin*, *lit-1/NLK *and *pop-1/TCF *is required to maintain proper timing of the spindle rotation of the ABar blastomere. It is possible that Bγ spindle orientation involves similar Wnt sub-pathways. Another possibility is that similar to Drosophila pI cell division, the Planar Cell Polarity pathway including *flamingo *and *strabismus *orients the Bγ spindle [53-55].

**Figure 4:**
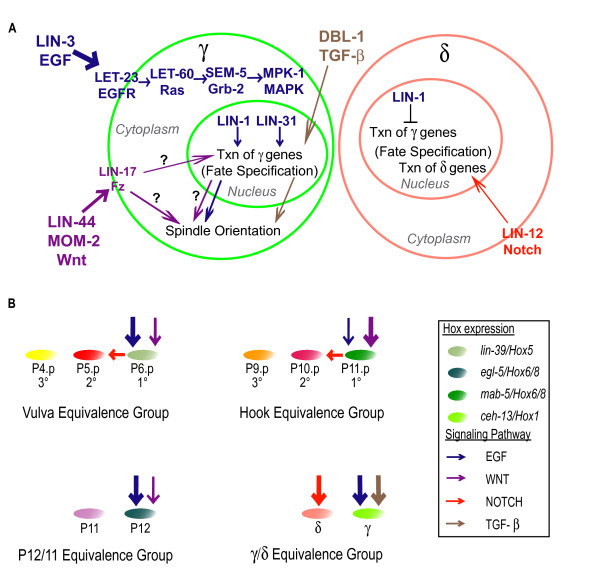
**Patterning of equivalence groups in *C. elegans *(A) Model for EGF, Wnt and TGF-β signaling during Bγ/δ specification**. The EGF and TGF-β pathways specify Bγ fate by regulating the transcription of target genes such as *ceh-13/hox1*. Wnt controls the axis of division of Bγ, possibly by orienting the mitotic spindle. POPTOP expression suggests Wnt may play a role in Bγ fate specification. (B) A comparison of the HCG, VPCs, P11/12 and Bγ/δ groups. EGF and Wnt signaling have different requirements relative to each other during the patterning of each equivalence group. This difference may account for the specificity of fate by both pathways induced in each group. In addition, Wnt signaling is required for Bγ division along the correct axis. Such a role for Wnt signaling has not been observed in the other equivalence groups. Another factor that may contribute to fate specification in each equivalence group is the use of a third pathway during patterning. TGF-β signaling by *dbl-1/dpp *is required to specify Bγ fate and does not appear to act during VPC and P12 specification, equivalence groups in which EGF signaling is the major inductive signal. Finally, downstream of the EGF and Wnt pathways, a different Hox gene is expressed in each equivalence group and required to specify fate within that group. One exception is *ceh-13/Hox1 *for which a functional role in Bγ fate specification has not been identified.

We propose that EGF and TGF-β activity specify Bγ by controlling target gene expression, while Wnt signaling acts to orient the Bγ mitotic spindle either by a transcriptional or non-transcriptional mechanism (Fig. 4A). Since the axis of division of Bγ in reduction-of-function mutants of components of the EGF pathway are mostly either Bγ-like (longitudinal) or Bδ-like (transverse), EGF signaling might control spindle orientation as a consequence of specifying the Bγ fate and may not directly target the cytoskeleton.

### Comparison of EGF and Wnt regulated Equivalence groups

Comparing the VPCs, HCG, P11/12 and Bγ/δ equivalence groups allows us to identify several similarities and differences that may explain how the same signaling pathways specify different fates in different equivalence groups. First, we have found a fourth example in which EGF/Ras signaling controls a Hox gene during fate specification in *C. elegans *(Fig. 4B). Although a role for *ceh-13/labial/Hox *in Bγ fate specification was not found (Additional File 1), we cannot rule out a requirement for *ceh-13 *because we were unable to assay terminal fates. Moreover, the positive regulation of *ceh-13/labial *by EGF signaling, which specifies the Bγ fate, and the conservation of Hox function in other cell fates regulated by EGF and Wnt signaling hints at a functional role for *ceh-13 *in Bγ: EGF and/or Wnt signaling upregulate *lin-39/Scr/Hox *to specify VPC fate [8], *egl-5/Abd-B/Hox9-13 *to specify P12 fate, and *mab-5/ftz/Hox *during hook development (see Introduction). Alternatively, *ceh-13 *may play a lesser role during fate specification.

One reason why *ceh-13*, as opposed to the other Hox genes, is positively regulated in Bγ may be due to TGF-β signaling, which also regulates *ceh-13 *expression and promotes the Bγ fate. Since the TGF-β signaling pathway does not appear to be involved in vulval and P12 specification, it probably does not act to regulate Hox genes in the VPCs and P11/12. Another possibility is that the specificity of Hox expression in the different equivalence groups may be a consequence of their developmental history. Prior to upregulation by EGF and/or Wnt signaling, *lin-39 *and *mab-5 *are already expressed in the VPCs and HCG, respectively. One possibility is that the presence of a different Hox gene in these two equivalence groups may bias the VPCs and the HCG to upregulate *lin-39 *and *mab-5*, respectively, in response to EGF and/or Wnt signaling. In the case of the Bγ/δ equivalence group, there is no prior expression of *ceh-13 *in either cell within the equivalence group. *egl-5 *is most probably not expressed in P11/12 before specification [56].

In contrast to the other equivalence groups, patterning of the Bγ/δ equivalence pair appears to involve competing signals from different cells outside the equivalence group to specify the Bγ and Bδ fates. Both fates are specified by other cells and do not appear to be required to specify each other. Therefore, there is no primary (1°) fate in the Bγ/δ equivalence group: isolated Bγ/δ precursors can adopt either the Bγ or Bδ fate [19,57]. In contrast, VPC and HCG specification utilize a sequential signaling mechanism to first specify the 1° fate, followed by lateral signaling to specify the 2° fate. Specification of the 2° fate usually requires the presence of the 1° fate. However, a graded signaling mechanism in which the EGF signal acts to specify the 1° and 2° VPC fates allows for isolated 2° fates. Within the P11/12 pair, the P12 fate is the 1° fate because an isolated P11/12 precursor always adopts the P12 fate, suggesting that there is no competing P11 fate specification signal. A sequential signaling mechanism does not appear to be used to specify the P11 fate, and there is no evidence for a model in which competing signals act to specify the P11 and P12 fates. Although the source of the EGF and Wnt patterning signals have not been determined for P12 specification, reduced EGF or Wnt activity results in the P11/12 pair adopting the P11 fate and intermediate P11/12 fates have not been observed. Neither a P11 fate specification signal nor a cell that promotes P11 fate has been identified.

Since several competing external signals specify the Bγ/δ pair and the axis of division of each fate in the Bγ/δ pair is distinct (transverse versus longitudinal), we were able to observe that fate specification and the angle of the division axis of Bγ appear to be separable. This appears similar to EMS blastomere development where orientation of the EMS mitotic spindle (by a non-transcriptional mechanism) and endoderm fate induction (by regulating gene transcription) are regulated by different Wnt subpathways [51,58]. Within the Bγ/δ pair, Wnt signaling controls the axis of division, possibly by orienting the Bγ mitotic spindle. By comparison, division axis defects are not observed in the other EGF and Wnt specified fates, P6.p (1° VPC), P11.p (1° HCGs) and P12 when EGF and/or Wnt signaling is compromised because the fate acquired by these cells either has the same mitotic spindle orientation as in wild-type or does not involve division. For example, the 3° VPC fate adopted by P6.p in *bar-1/β-catenin *mutants results in P6.p dividing once along the same axis that it would have divided if it had adopted the 1° fate. Further study of each equivalence group will allow us to determine other generalities of how the same signals are used to specify different cell fates and to determine how the same signals interact differently to specify fate.

## Conclusions

We provide evidence that *ceh-13/labial/Hox1 *expression in Bγ is regulated by the EGF-Ras pathway and downstream factors *lin-1/ETS*, *lin-31/Forkhead *and *sur-2/Med23*. We also show that Wnt signaling is required for proper Bγ division, and we propose that the Wnt receptor *lin-17 *and the Wnts *lin-44 *and *mom-2 *help orient the Bγ mitotic spindle. Finally, we show that *dbl-1/dpp *is not required for VPC and P12 specification. Therefore, our results suggest that another reason for fate specificity among EGF-regulated equivalence groups is the use of a third pathway e.g. TGF-β signaling in the case of Bγ fate specification.

## Methods

### Genetic methods and strains

Strains were grown at 20°C as described in Brenner (1974), unless otherwise indicated [59]. All strains used contain the *him-5(e1490) *mutation [60] which has been omitted from the following description of the strains used:

PS21: *let-23(sy1)*, PS4807: *syIs145 *[*ceh-13*::GFP] (described below), PS4814: *syIs145*; *let-60(n1046gf)*, PS5000: *syIs145*; *lin-15(e1763)*, PS5014: Ex[HS::*lin-3*; *pha-1*(+); *myo-2*::GFP], PS5026: *syIs145*; *lin-1(e1777)*, PS5031: *syIs145*; *sem-5(n1619)*, PS5032: *syIs145*; *let-60(n2021)*, PS5087: *syIs145 lin-31(bx31)*, PS5101: *syIs145 lin-31(n301)*, PS5193: *lin-17(n698)*; *syIs145*, PS5207: *syIs145 cwn-1(ok456); egl-20(n585)*, PS5208: *syIs145*; *lin-1(n1790gf)*, PS5256: *lin-44(n2111)*; *syIs145*, PS5501: *syIs145*; *dbl-1(wk70)*, PS5628: *syIs197 *[HS::*lin-3C*, *myo-2*::dsRed, *pha-1*(+), KS(+)], PS5667: *dbl-1(wk70)*; *sem-5(n1779)*, PS5869: *syIs145*; *syIs197*, PS5870: *syIs145 lin-31(n301)*; *syIs197*, PS5872: *syIs145*, *lin-1(n1790gf)*; *syIs197*, PS5879: *dbl-1(wk70); sem-5(n1779)*, PS5881: *lin-17(n698)*; *syIs188*, PS5889: *sem-5(n1779)*, PS5896: *lin-44(n2111)*; *syIs188*, PS5905: *let-23(sy97) syIs145*. *let-23(sy1)*; *dbl-1(wk70) *was constructed using PS21 and PS5501.

PS4807 contains the *ceh-13*::GFP integrated transgene *syIs145 *that was obtained by microinjection of pMF1 [24] at 10 ng/μl, pBS at 20 ng/μl and *unc-119*(+) at 40 ng/μl into *unc-119(ed4)*; *him-5(e1490) *mutant animals.

### Analysis of strains carrying the *ceh-13::GFP integrated transgenes*

GFP expression was analyzed using Nomarski optics and fluorescence microscopy and viewed using a Chroma Technology High Q FITC filter set. Still images were captured with a Hamamatsu digital camera and Improvision Openlab software version 5.02.

*ceh-13*::GFP expression was scored in the mid-L3 stage when the B.a progeny had moved into their final positions.

### Laser Ablations

U and F cell ablations were performed as previously described [19].

### Heat-shock induction of *HS::lin-3 transgene*

Plates with well-fed animals were sealed with parafilm and floated in a 33°C water bath for 1 hour to induce the heat-shock response. Animals were scored 3 to 6 hours later.

## Authors' contributions

AS participated in the design of the study, carried out all experimental procedures and drafted the manuscript. PWS conceived of the study and helped to draft the manuscript. Both authors have read and approved the final manuscript.

## Supplementary Material

Additional file 1 **Supplemental Information**. Transcription Factors Downstream of or in Parallel to the EGF pathway and effects of *ceh-13 *RNAi on Bγ division axis and *ceh-13*::GFP expression in Bγ Click here for file
