# Supplementary material for: The roles of EGF and Wnt signaling during patterning of the C. elegans Bγ/δ Equivalence Group
Source: BMC Dev Biol. 2009 Dec 31;9:74. doi: 10.1186/1471-213X-9-74 (PMC2813230; doi:10.1186/1471-213X-9-74)
Supplement: Additional file 1 — Supplemental Information. Transcription Factors Downstream of or in Parallel to the EGF pathway and effects of ceh-13 RNAi on Bγ division axis and ceh-13::GFP expression in Bγ [file 1471-213X-9-74-S1.DOC]

**Supplemental Information**

**Table S1. Transcription factors that were not required for *ceh-13*::GFP expression**

| Genotype^a^ | **n** | ***ceh-13*::GFP**^b^ **in Bγ**  **(%)** |
| --- | --- | --- |
| Intact, wild type | 41 | 100 |
| *eor-1(ok1127)*^b^ | 33 | 100 |
| *eor-1(cs28null)* | 37 | 97.3 |
| *eor-2(cs42rf)* | 32 | 93.75 |
| *egl-5* RNAi^c^ | 20 | 100 |
| *lin-39* RNAi^c^ | 20 | 100 |

^a^ All strains contained *him-5(e1490)*.

^b^ The *ok1127* allele was made by the OMRF Knockout Group and has an estimated deletion of about 1.2 kb.

^c^ Feeding RNAi was carried out using clones from the Ahringer Library.

***ceh-13* RNAi does not affect Bγ division axis or *ceh-13*::GFP expression in Bγ**

Because *ceh-13/labial/Hox1* was expressed in Bγ and EGF signaling regulates Bγ fate specification, we wanted to test whether *ceh-13* was required for Bγ fate specification. However, *ceh-13(null)* mutations cause embryonic lethality in the majority of animals and only a small percentage of sickly survivors manage to persist to adulthood. Stoyanov et al. (2003) reported that viable *ceh-13(null)* males do not have any defects in mating, suggesting that spicule formation is normal in these mutants. We also observed that viable *ceh-13(null)* males have wild-type spicules (n=3). Because the survivors are not healthy, it is difficult to perform lineage analysis.

To bypass the requirement for *ceh-13/labial* during embryonic development, we made a heat-shock inducible *ceh-13* exon 1 hairpin RNAi construct where the hairpin was cloned into the heat-shock vector pPD49.83. Heat-shock three to five hours before the first Bγ division had no effect on the axis of division: Bγ divided longitudinally in all heat-shocked HS::*ceh-13* RNAi animals and *ceh-13*:GFP expression was normal (n=16). Interestingly, we observed that there was ectopic expression of *ceh-13*::GFP in the other B.a progeny in these animals (Supplemental Fig. S1): *ceh-13*::GFP was expressed in Bδ, Bζ and Bα in about 20% of animals and in Bβ and Bε in about 50% of animals (n=15). These results suggest that *ceh-13* is present at very low levels to negatively autoregulate its own expression in the other B.a progeny. The effects of HS::*ceh-13* RNAi on Bα, Bβ, Bδ, Bε and Bζ indicate that the construct is able to reduce *ceh-13* levels. However, it is still possible that *ceh-13* activity is not sufficiently lowered by the RNAi construct. Therefore, further analysis will be necessary to determine whether *ceh-13* is required to specify the Bγ fate.

**
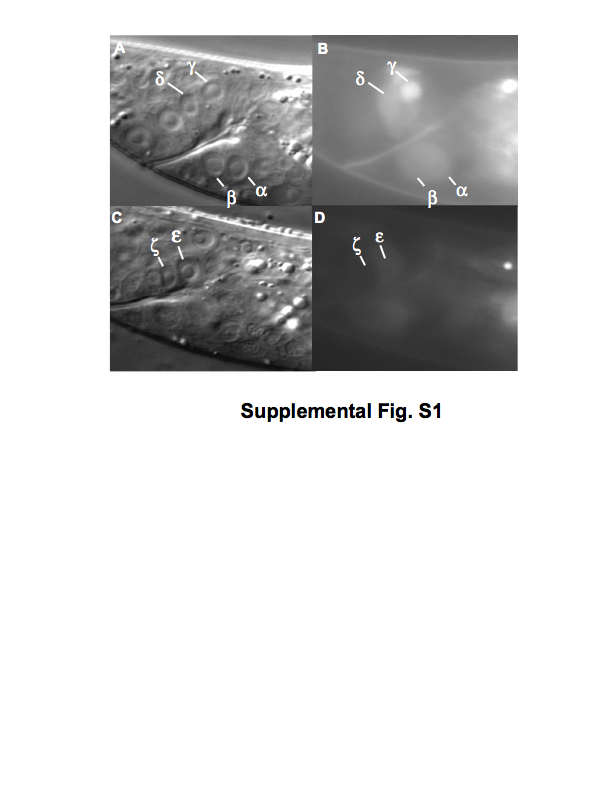
**

**Supplemental Figure S1.** Heat-shock inducible *ceh-13* RNAi caused ectopic *ceh-13*::GFP expression in B.a progeny. (A-B) Mid-L3 male. Ectopic expression was observed in Bα, Bβ and Bδ. (C-D) Mid-L3 male. Ectopic expression was observed in Bε. Left lateral views.
